# Adapting the WHO recommendations on health worker roles for safe abortion to a country setting: A case study from India

**DOI:** 10.1002/ijgo.13001

**Published:** 2020-07-31

**Authors:** Vinoj Manning, Bela Ganatra, Medha Gandhi, Anchita Patil

**Affiliations:** ^1^ Ipas Development Foundation New Delhi India; ^2^ UNDP‐UNFPA‐UNICEF‐WHO-World Bank Special Programme of Research, Development and Research Training in Human Reproduction (HRP) Geneva Switzerland; ^3^ Independent Public Health Consultant Delhi India

**Keywords:** Advocacy, Comprehensive abortion care, India, Medical Termination of Pregnancy Act 1971, Task‐sharing

## Abstract

In 2015, the World Health Organization (WHO) published a guideline on the role of health workers in providing safe abortion and postabortion contraception, with evidence‐based recommendations on the range of providers who can perform interventions to provide safe abortion, postabortion care, and postabortion contraception. The WHO guideline is global in nature and must be contextualized to individual country settings. The present paper compares the scenario in India, including the legal and policy frameworks, with the WHO guidelines. It provides legal and policy recommendations that are needed to improve access to comprehensive abortion care in India, with a focus on expanding the provider base. The process used to develop these recommendations was a combination of empirical evidence gathering and multistakeholder consultations. An outcome of this exercise was a policy brief entitled “Improving access to comprehensive abortion care in India with focus on expanding provider base,” which is used as an advocacy tool.

## Abstract 2

The process followed by multistakeholders to develop evidence‐based country‐specific recommendations focusing on expanding the provider base using WHO guidelines on the role of health workers to provide comprehensive abortion care.

## 1 Background

The Medical Termination of Pregnancy (MTP) Act was enacted in India in 1971 to provide the legal framework for abortion services in the country.^1^ Despite this and after decades of legalization of abortion services, India has a long way to go to ensure universal access to comprehensive abortion care services. Comprehensive abortion care refers to the provision of safe, high‐quality, woman‐centric abortion services and includes abortion, postabortion care, and postabortion contraception.

A recent study estimated that the incidence of induced abortions in India was 15.6 million in 2015.^2^ Of these, only 3.4 million (22%) took place in a facility setting using either surgical or medical methods. A substantial 73% of the total—about 11.5 million abortions—took place outside a facility using medical methods. One reason for this scenario is the lack of access to facilities registered under the MTP Act to provide abortion services—such facilities are not only fewer than required but are also inequitably distributed.^3^ The other critical barrier is the shortage of trained and “certified” providers of abortion.^4^ The MTP Act permits only allopathic doctors—obstetrician/gynecologists (ob/gyns) or general physicians who in India possess the Bachelor of Medicine, Bachelor of Surgery (MBBS) degree with defined experience in this field—to provide abortion services. However, data for the year 2015–2016 reported only 1292 ob/gyns in public health facilities compared with 5510 required specialists, translating to a shortfall of over 76%.^5^ It is estimated that there are around 35 000 ob/gyns in the private sector but most of them are in urban areas. While there is also a shortfall in other health worker cadres, the same dataset reveals much higher availability of nurses and auxiliary nurse midwives (ANMs), both in absolute numbers and as a proportion of the total requirement in public health facilities. For example, there were over 69 000 nurses and about 220 000 ANMs posted at government‐owned facilities, with a shortfall of only 20.5% and 5.3%, respectively.^5^

In 2015, the World Health Organization (WHO) published the guideline “Health worker roles in providing safe abortion care and postabortion contraception”^4^ (henceforth, the WHO 2015 guideline), which provides evidence‐based recommendations on the range of healthcare providers who can effectively and safely perform various interventions for provision of safe abortion and postabortion care. This guideline complements WHO's technical and policy guideline on safe abortion,^6^ which describes evidence‐based interventions for comprehensive abortion care along with the required legal and policy frameworks to ensure ease of access to these services for women. The WHO guideline is global in nature and must be adapted to individual country settings based on the local conditions, including the legal and policy frameworks.

Following the launch of the WHO 2015 guideline, the Ipas Development Foundation (IDF), in collaboration with the UNDP‐UNFPA‐UNICEF‐WHO‐World Bank Special Programme of Research, Development and Research Training in Human Reproduction (HRP) at the Department of Reproductive Health and Research, WHO, undertook an exercise to compare the existing scenario in India against the recommended global norms and use the review findings to suggest legal and policy changes for expanding the role of health workers to provide abortion and postabortion care.

The aim of the present paper was to highlight the process used to develop the required legal and policy recommendations, including the challenges faced, and to share the final recommendations that emerged as the key outcome of the process.

## 2 Process Used to Develop the Recommendations

A two‐stage process was followed, as depicted in Figure 11. Stage one involved in‐depth review and documentation of the Indian scenario,^7^ including: (1) comparing health personnel cadres that are available in India, matching these with the definitions provided by WHO in its 2015 guideline (Table 11), and researching the roles they are allowed to play based on available data; (2) conducting an in‐depth review of legal, policy, and other data sources (Table 22) to determine the Indian legal and policy scenario regarding the eligibility of various health personnel cadres to provide abortion and related services; and (3) comparing it with the WHO recommendations and mapping the gaps between the two to develop a list of potential recommendations for legal and policy changes required in India to bring the country situation on par with the WHO recommendations.

**Figure 1:**
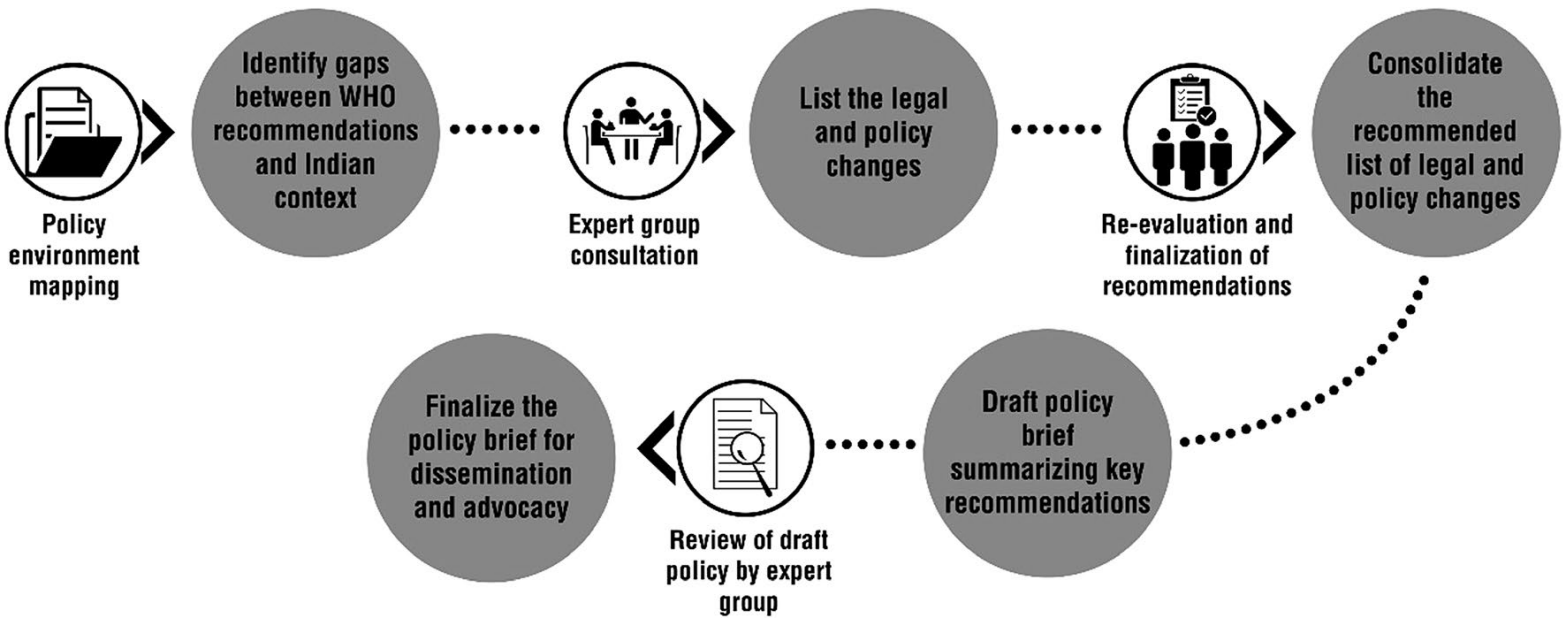
Process for developing a policy brief with legal and policy recommendations on expanding the health provider base to improve access to abortion care.

**Table 1:** Matching health provider types as listed by WHO with health provider cadres in India

Serial no.	Health provider cadre as listed by WHO	Comparative health provider cadre in India
1.	Lay health worker	Accredited Social Health Activist
2.	Pharmacy worker	Pharmacy worker
3.	Pharmacist	Pharmacist
4.	Doctor of complementary systems of medicine	AYUSH doctor
5.	Auxiliary nurse/auxiliary nurse midwife	Auxiliary nurse midwife
6.	Nurse	Nurse
7.	Midwife	No matching cadre
8.	Associate/advanced associate clinician	No matching cadre
9.	Nonspecialist doctor	MBBS/Allopathic doctor
10.	Specialist doctor	Obstetrician/gynecologist

Abbreviations: AYUSH, Ayurveda, yoga, naturopathy, Unani, Siddha, or homoeopathy doctors; MBBS, Bachelor of Medicine, Bachelor of Surgery.

**Table 2:** List of Indian legal and policy documents, websites, and data sources related to provision of abortion services reviewed

Comprehensive abortion care‐related documents	Other technical and policy documents	Documents related to cadre matching
The Medical Termination of Pregnancy (MTP) Act, 1971, along with The Medical Termination of Pregnancy Rules, 2003 and The Medical Termination of Pregnancy Regulation, 2003^1^ CAC Training and Service Delivery Guidelines, 2010^14^ CAC Operational Guidelines, 2014^15^ CAC Provider's Manual, 2014^16^ MMA Handbook, 2016^17^ Postabortion Family Planning Technical update, 2016^18^ Operational Guidelines—Postabortion Family Planning 2016^19^	Pregnancy and delivery care guidelines^8^, ^9^ Family planning guidelines on female sterilization,^20^ Intra Uterine Contraceptive Device insertion,^21^, ^22^ injectable contraceptives^23^ Drug Controller General of India recommendations on drug categorization and usage	Course curricula for AYUSH doctors (Ayurveda,^10^ Unani,^11^ Siddha,^12^ homeopathy^13^), nurses, ANMs, pharmacists, and ASHAs Course curriculum and competencies defined by the International Confederation of Midwives^24^ Rural health statistics, websites of professional councils, press articles (for data on numbers of providers of different cadres)

Abbreviations: CAC, comprehensive abortion care; MMA, medical methods of abortion; ANM, auxiliary nurse midwife; ASHA, Accredited Social Health Activist.

For the second stage, the review was presented to and discussed with experts at two consultative meetings; each meeting was held with a different primary purpose and methodology. The first meeting comprised a smaller group of 16 “core experts” and was an intense exercise with a two‐fold purpose: (1) finalizing the gap analysis (Figure 22) based on the desk review done by IDF; and (2) prioritizing the potential recommendations that emerged from the desk review. This was done based on the potential impact of the recommendation on improving access to comprehensive abortion care as well as the feasibility of bringing about that legal or policy change. The second meeting comprised, in addition to the participants from the first meeting, representatives from a wider range of stakeholder groups, including legal experts and representatives from civil society and women's groups. The purpose of this group was to re‐evaluate and ratify the recommendations prioritized by the first group and bring in broad‐based perspectives from different stakeholders.

**Figure 2:**
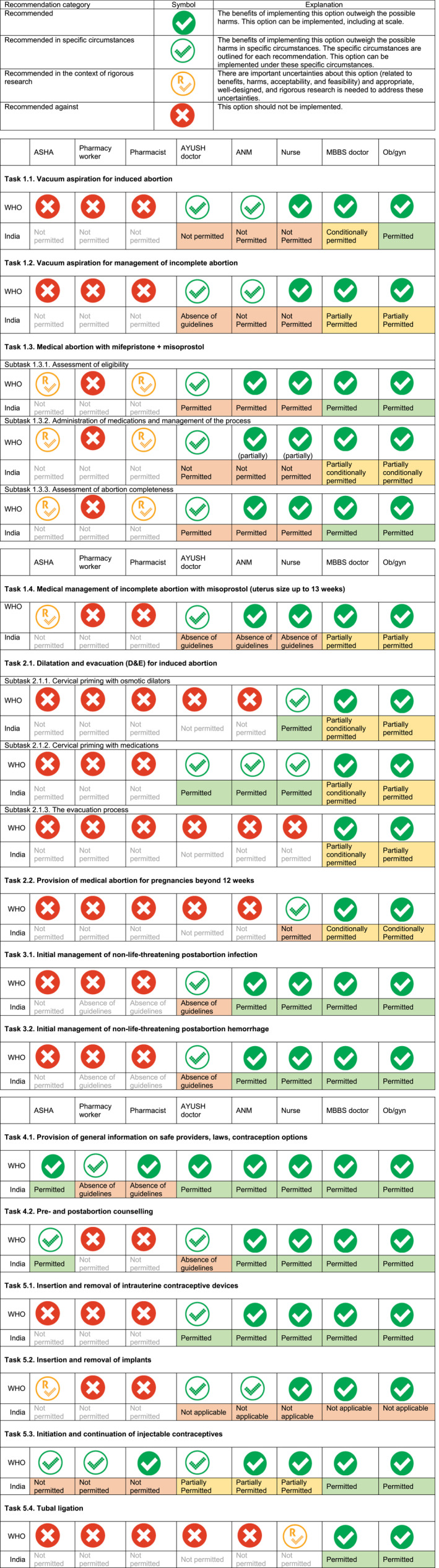
Comparison between the WHO recommendations and the Indian legal and policy landscape on health worker eligibility for delivery of comprehensive abortion care services (see comparison symbols key). Abbreviations: ASHA, Accredited Social Health Activist; AYUSH, Ayurveda, yoga, naturopathy, Unani, Siddha, or homoeopathy; ANM, auxiliary nurse midwife; MBBS, Bachelor of Medicine, Bachelor of Surgery; Ob/gyn, obstetrician/gynecologist. Figure reproduced with permission from Ipas Development Foundation.

The recommendations finalized at the second meeting (Table 33) were translated into a policy brief^25^ to be shared with the government and other advocates for improving access to comprehensive abortion care services, with special focus on expansion of the provider base. The recommendations focused primarily on task‐sharing for first‐trimester abortions.

**Table 3:** Key recommendations that emerged from the consultative process

No.	Current status in India	Recommended/desired status	Legal/policy change required	Potential impact
1.	First‐trimester abortions			
a.	Only specialist allopathic doctors (ob/gyn), or doctors with special training in abortion provision and/or experience in ob/gyn can perform induced abortions	Additional cadres such as AYUSH doctors, nurses, and ANMs be permitted to conduct abortions^a^	Amendment of the law (MTP Act), followed by related revisions to the MTP Rules and policy documents	Increase in number of abortion providers by adding thousands of potentially trainable providers for provision of abortion services
b.	Public sector primary healthcare facilities are permitted to offer abortion services for pregnancies up to 8 wk gestation only	Primary healthcare facilities to offer abortion services for the complete first trimester (12 wk of gestation)	Revisions to the Government oi India CAC Operational Guidelines	Improved access to first‐trimester abortions (up to 12 wk) by making them available in an increased number of facilities and therefore closer to women's homes
2.	Incomplete abortion			
a.	Policy guidelines do not permit nurses and ANMs to manage incomplete abortions and are silent about the role of AYUSH doctors for the same	All cadres of health personnel available at referral facilities, including AYUSH doctors and nurses, be trained and permitted to manage incomplete abortions	Revisions to Government of India CAC technical and training guidelines	Timely management of all incomplete abortions, by skilled persons using appropriate technologies, can prevent the occurrence of secondary life‐threatening complications such as infections and hemorrhage
b.	Policy documents restrict the definition of incomplete abortion to only those following an induced medical abortion	The definition of incomplete abortion to include spontaneous onset incomplete abortions as well as those following surgical methods		
c.	Policy documents restrict the management of incomplete abortions to the use of vacuum aspiration only; misoprostol use is restricted to very specific circumstances^b^	Inclusion of both vacuum aspiration and misoprostol administration as effective and safe methods for management of incomplete abortions		
3.	Other postabortion complications			
a.	Policy documents are silent about the role of AYUSH doctors in the management of postabortion complications such as hemorrhage and infections	AYUSH doctors also be permitted (like nurses and ANMs) to offer lifesaving care for postabortion complications like hemorrhage and infections, including the administration of IV fluids and parenteral antibiotics	Revision of Operational Guidelines	AYUSH doctors are available at (and in many places are in charge of) 40% of the primary healthcare facilities; permitting them to provide these services will reduce morbidity and mortality due to postabortion complications
4.	Medical abortion			
a.	Law and policy documents restrict the use of MMA until 7 (or 9^c^) weeks only	Permit the use of MMA for inducing abortion until the legally allowable gestational age for termination of pregnancy (presently 20 wk)	Amendments to the Rules of the MTP Act and the DCGI's guidance, followed by related revisions to the policy documents	Extending the use of MMA in terms of gestational age will increase the technology options available to women seeking abortion beyond 7 (or 9^c^) weeks of gestation
5.	Self‐management of first‐trimester abortions using MMA			
a.	The technical guidelines on MMA require the woman availing an induced abortion through MMA to visit the facility thrice: for initial assessment and administration of mifepristone; for administration of misoprostol; and for assessing completeness of abortion	If the woman is provided accurate information, she can safely and effectively self‐administer the dose of misoprostol at home; thereby eliminating the second visit to the facility/provider	Revisions to Government of India's technical guidelines related to MMA	Reduction of one facility level visit increases convenience for women seeking MMA, by saving time and incidental costs
6.	Information and counselling			
a.	Policy documents are silent on the role of the pharmacist and pharmacy workers in the provision of abortion‐related information	Pharmacists and pharmacy workers be permitted and trained to provide basic abortion‐related information such as its legality, where to go, the eligible providers etc.	Revision of the Operational Guidelines followed by incorporation of these additional cadres in the Government of India's CAC technical and training guidelines	With many women seeking to buy MMA drugs directly from pharmacies, pharmacists and pharmacy workers often become the first point of contact for women seeking abortion. They can play a crucial role in offering correct abortion‐related information to women in need

Abbreviations: AYUSH, Ayurveda, yoga, naturopathy, Unani, Siddha, or homoeopathy doctors; ANM, auxiliary nurse midwife; MTP, medical termination of pregnancy; CAC, comprehensive abortion care; MMA, medical methods of abortion; DCGI, Drug Controller General of India.

a From a feasibility and impact viewpoint, the experts recommended initiating the change with permitting these additional cadres to provide abortion using medical methods only; permission to use vacuum aspiration for abortion may be added later.

b Misoprostol for management of incomplete abortion is discussed in the documents only in cases of failed MMA. Even in this, it is restricted to specific cases where the gestational sac is visible on an ultrasound but is not viable.

c While the Rules of the MTP Act mention 7 weeks as the maximum gestation until which MMA can be used to induce abortion, the permission by the DCGI for using the mifepristone plus misoprostol combi‐pack for abortion is until 9 weeks of gestation.

It is important to note that in mapping health worker eligibility in India, the terms “partial” and “conditional” eligibility have been used. Partial eligibility refers to contexts where the health worker is eligible to perform the task but other conditions (such as gestational age limits) do not allow the worker to perform the task to the whole extent of WHO's technical recommendations. Similarly, conditionally permitted is used in situations where the health worker's eligibility to perform the task is limited through policy/technical guideline conditionalities, such as additional site approvals.

### 2.1 Documents generated through the process

Papers documenting the in‐depth analysis were also developed. The documents, “Expanding Provider Base for Safe Abortion in India: Policy Gaps”^7^ (which describes the situation analysis of India's legal and policy scenario concerning the eligibility of health worker cadres to perform various abortion‐related tasks, with detailed references to the various documents reviewed), and “Expanding Provider Base for Safe Abortion in India: Policy Actions”^26^ (which is a more concise and easy‐to‐read version of the Indian policy analysis document), along with the final policy brief^25^ are available in the public domain and can be used by advocates working to make legal and policy changes in the abortion context, health and human rights experts, law and policy makers working on abortion‐related issues, as well as academics and researchers studying the abortion scenario in other countries.

## 3 Challenges and Lessons Learned from the Process

While the process of comparing the national scenario with the global guideline and drawing recommendations should have been a relatively straightforward process, we faced several challenges, both expected and unexpected.

The purpose of the exercise was to develop recommendations and ensure that they are acceptable to a wide range of stakeholders, including those who have a role to play in implementing the recommended changes, such as the different cadres of health service providers. To achieve this we included varied stakeholders in our consultative meetings, including ob/gyn specialists, AYUSH (Ayurveda, yoga, naturopathy, Unani, Siddha, or homoeopathy) doctors, nurses, government decision makers, public health experts, researchers, legal experts, advocacy experts, civil society leaders, and representatives of multilateral organizations. There were many advantages to following this inclusive process, which: (1) ensured ownership of the process and the finalized recommendations by representatives of key stakeholder groups; (2) enabled sharing of varied perspectives on the issues, making the recommendations balanced and well‐thought through; (3) provided inputs on the need and feasibility of the changes recommended by the people who were engaged, in one way or another, with abortion care; and (4) initiated discussions on comprehensive abortion care‐related issues with and between the different stakeholders, both within and beyond the meetings, which will be required to bring the recommended legal and policy changes to fruition.

The challenge in engaging with such a large group was merging the divergent opinions into a common consensus. The following management steps and tools were used to arrive at a consensus: (1) sharing detailed documentation of the desk review findings (and adding on the discussions and decisions taken at the first meeting) in the “Expanding Provider Base for Safe Abortion in India: Policy Gaps”^7^ and “Expanding Provider Base for Safe Abortion in India: Policy Actions”^26^ before the scheduled meeting to ensure that all participants were on the same page in terms of the initial analysis done; (2) dividing the large team of experts into smaller working groups to allow for in‐depth discussions, followed by sharing of their decisions through a “plenary”; and (3) sharing predesigned templates with the working groups for reporting the discussions and decisions. This not only kept the decisions focused on the purpose of the meeting but also helped consolidate opinions to develop recommendations for the final policy brief.

In addition to the comprehensive abortion care‐related Indian legal and policy documents (about seven) we also reviewed documents related to other technical themes; for example, management of spontaneous abortions was not covered in the comprehensive abortion care documents but in the technical guidelines for skilled birth attendants—the former focused on induced abortions only. The cadre‐matching exercise required us to study the course curricula of many healthcare provider cadres in India to ensure comparability with the WHO definitions. In addition, the emergence of new documents and changes in policies during the process added to the list of documents to be reviewed and required revisions to the comparative analysis and final recommendations. For example, the operational guidelines for postabortion family planning^19^ were updated just before the second expert group meeting, which necessitated updating the mapping charts and removing some recommendations for policy change as the required change had already been brought about by the government.

Another challenge encountered was the lack of information on the roles of certain cadres in India. The Indian Comprehensive Abortion Care Operational Guidelines^15^ do not mention abortion‐related tasks for cadres such as AYUSH doctors, pharmacy workers, and pharmacists. Besides this, the Indian documents do not differentiate between cadre eligibility for performing a complete task compared with performing just a component within the task (what the WHO guideline refers to as a “subtask”). Many policy documents, such as the postabortion family planning technical guidelines,^18^ did not specify the health worker cadre(s) they were meant for. In the initial desk review, this lack of information was interpreted as lack of eligibility of that cadre to perform the tasks and subtasks. While in most cases the experts agreed with this assessment, in a few cases the experts opined that in situations where cadres are not specified, it can be considered that they are permitted to provide the service. For example, the law (MTP Act) permits only allopathic (MBBS) doctors with a qualification and/or experience in ob/gyn to conduct abortions. The initial review interpreted this to mean that nonphysician cadres were not permitted to perform even the related subtasks, such as cervical dilation (using osmotic dilators or medications) prior to a dilatation and evacuation. The experts, however, felt that the law was applicable to the actual provision of abortion, that is, the evacuation process only, and that in a facility setting the law did not restrict other cadres from performing the preparatory subtasks. They argued that a relatively restrictive interpretation of the law or guidelines reinforced an artificial barrier of service provision and could hinder the path for expansion of the provider base. In situations where the guidelines were silent on certain components of abortion care, the experts agreed to take a nonconservative position.

A prominent example of conflicting information in different documents is the gestational age up to which medical methods of abortion can be used. While the rules of the MTP Act restrict the use of medical methods up until only 7 weeks of gestation, the office of the Drug Controller General of India (DCGI) has approved the use of the medical methods combi‐pack (mifepristone and misoprostol) until 9 weeks of gestation, creating confusion regarding the legal status. To add to the uncertainty, the medical methods of abortion handbook^17^ on one hand mentions the gestational age restrictions as specified in both the MTP Act and by the DCGI, but on the other hand also describes the process for using medical methods for the complete first trimester. The review notes these differences as a gap and recommends matching information in various documents and expanding the gestational age for provision of medical methods of abortion to cover the complete first trimester. In another example, while the WHO 2015 guideline makes a clear distinction between the terms “information provision” and “counselling,” this differentiation did not exist in Indian policy documents where the word “counselling” was used to mean either information provision or counselling. For example, according to these policy documents even the lay worker, like the Accredited Social Health Activist (ASHA), was expected to “counsel” women on comprehensive abortion care.

While the focus of the process was on assessing gaps and developing recommendations related to expansion of the provider base for provision of comprehensive abortion care, the review revealed gaps that went beyond provider eligibility, which necessitated recommendations beyond expansion of the provider base to ensure improved access to comprehensive abortion care services. For example, while the MTP Act permits registered allopathic (MBBS) doctors to perform first‐trimester abortions, there is a facility‐level restriction in the Government of India's operational guidelines^15^; doctors posted at primary health centers are allowed to provide abortion only until 8 weeks of gestation, while doctors with similar qualifications posted at higher facilities can induce abortions for the complete first trimester. The gestational age restriction of provision of medical methods of abortion (mentioned above) was also an issue delinked to provider cadre, but one that impinged on their ability to provide the complete bandwidth of services as recommended in the WHO guideline. The final recommendations went beyond mere expansion of the provider base for provision of services to also include removal of legal and policy restrictions that reduce access to abortion care.

Another challenge was the different interpretation of the law by the medical and legal experts, which arose in the second meeting. The medical experts believed a “liberal” interpretation of the law should be taken concerning eligibility to perform various subtasks related to abortions. According to them, the MTP Act defined eligible providers only for provision of the actual abortion procedure. Given this view, there is no restriction on any cadre for provision of other subtasks, such as assessment of eligibility for medical methods of abortion or assessment of abortion completeness, and therefore IDF's earlier assessment that AYUSH doctors, nurses, and ANMs are not permitted to perform these tasks should be revised. They also felt that interpretation of the Act should be more expansive than restrictive, as that may be the first step toward expanding the provider base for comprehensive abortion care. In contrast, the legal experts felt that a more restrictive interpretation should be adopted (as done by IDF in the initial background note and accepted by the first group of experts) to safeguard the interests of the providers. Should an issue arise regarding “mismanagement” of a case involving nonphysician health personnel, the health personnel might be found at fault. As such a case has never arisen, there is no precedent set in court to give a sense of which way the judgment would go.

## 4 Discussion

The process used a combination of empirical evidence gathering and multistakeholder consultations. The legal and policy review of comprehensive abortion care in India with reference to the WHO guideline resulted in legal and policy recommendations that are needed to improve access to abortion care in India, with a focus on expanding the provider base.

The curated evidence in the form of the policy synopsis and the policy brief^25^ are valuable resources available in the public domain, which abortion and public health advocates, law and policy makers, and academics and researchers may find useful. The process brought together stakeholders from multiple domains and led to an initial discussion on the issue, which will support the advocacy efforts that are needed to ensure that the proposed recommendations are adopted by the government and changes are made to the abortion act and comprehensive abortion care policies.

Agencies working in the area of abortion in India have used the policy brief and the “Expanding Provider Base for Safe Abortion in India: Policy Actions”^26^ document to develop advocacy materials and inform the media on the need for policy change to expand the role of health workers for abortion care provision. The process documents could provide useful guidance for other countries undertaking a task‐sharing exercise based on the WHO recommendations.

## Author Contributions

VM and BG conceptualized the study design and methodology. MG led the study implementation and process of organizing and facilitating the two expert group meetings and ensured incorporation of comments from experts. AP led the process of writing the manuscript and developed the first draft. All authors contributed to subsequent revisions and development of the final version.

## Conflicts of Interest

The authors have no conflicts of interest.
